# A combined approach of simulation-based “debriefing with good judgment” and case-based learning to enhance clinical thinking in Chinese residents

**DOI:** 10.3389/fpubh.2025.1718961

**Published:** 2026-02-04

**Authors:** Wenming Shao, Xin Cheng, Wanjie Gu, Yu Yan, Hiu Liu, Jialin Zhang, Li Zhang, Haiyan Yin, Mingya Zhang

**Affiliations:** 1 Department of Emergency, The First Affiliated Hospital of Jinan University, Guangzhou, China; 2 School of Medicine, Jinan University, Guangzhou, China; 3 Department of Intensive Care Unit, The First Affiliated Hospital of Jinan University, Guangzhou, China; 4 Functional Experimental Teaching Center, School of Basic Medicine and Public Health, Jinan University, Guangzhou, China; 5 Laboratory Construction Section, Office of Lab and Facility Management, Jinan University, Guangzhou, China; 6 Clinical Skills Comprehensive Training Center, The First Affiliated Hospital, Jinan University, Guangzhou, China

**Keywords:** debriefing with good judgment (DwGJ), case-based learning (CBL), medical simulation, education, Chinese residents

## Abstract

**Objective:**

Clinical reasoning is a fundamental and critical competency for physicians. However, how to effectively help resident trainees to develop and enhance this ability remains a challenge for medical instructors. In this study, a teaching approach that combines simulation-based “Debriefing with Good Judgment” (DwGJ) with Case-Based Learning (CBL) was employed to improve the clinical reasoning among standardized training residents. The effectiveness of teaching strategy was evaluated.

**Methods:**

This study was designed and conducted as a randomized controlled educational trial. A total of 70 residents at the Clinical Skills Training Center of the First Affiliated Hospital of Jinan University, Guangzhou, China, were recruited in the 2023–2024 academic year. These residents from Internal Medicine, Surgery, Obstetrics and Gynecology, Pediatrics, Emergency Medicine, and Intensive Care Medicine, were randomly assigned to either a control group or a DwGJ group. There were 35 participants in each group. The control group received conventional CBL instruction, practicing skills within a clinical scenario, while the DwGJ group incorporated a DwGJ session following hands-on practice session. Upon course completion, participants evaluated the instructor and course, their clinical reasoning competencies (including clinical thinking, and systematic thinking, and evidence-based thinking) were surveyed, and theoretical and practical examination scores were compared.

**Results:**

There was no significant difference between two groups in instructor evaluation. However, the DwGJ group reported significantly higher satisfaction with the course. Moreover, the DwGJ group outperformed the control group in all subcomponents of clinical reasoning, including critical thinking, systematic thinking, and evidence-based thinking. Both the theoretical test scores and skill scores were significantly higher in the DwGJ group (64.40 ± 13.22 and 80.54 ± 7.4, respectively) compared to the control group (52.34 ± 18.42, *p* = 0.02 and 72.32 ± 7.6, *p* < 0.01, respectively).

**Conclusion:**

The integration of simulation-based DwGJ with CBL demonstrates more efficacy in enhancing resident trainees’ clinical reasoning abilities, through fostering their critical, systematic, and evidence-based thinking.

## Introduction

Medical resident training is globally recognized as an essential component of continuing medical education, aiming to cultivate novice physicians into competent, independent practitioners. Therefore, residency training is crucial to the quality and advancement of healthcare services. The goal and expected outcome of residency training is for residents to acquire competencies. For physicians, competence has been acknowledged as “the habitual and judicious use of communication, knowledge, technical skills, clinical reasoning, emotions, values, and reflection in daily practice for the benefit of the individual and community being served” (1, 2). Among these essential attributes, clinical reasoning represents a higher-order cognitive skill. It refers to the capacity to efficiently assess, interpret, and manage a patient’s condition within a limited time frame and with minimal resources, aiming to optimize clinical outcomes. Cultivating this ability in medical trainees requires addressing several common challenges, such as disorganized thinking, delayed decision-making, and a lack of coherence and logical structure in their reasoning processes. For instance, during medical history taking, students often collect patient information in a superficial or disorganized manner, which may lead to omissions and increase the likelihood of misdiagnosis. Similarly, during clinical observation, learners may focus on a specific stage of a patient’s illness while neglecting the overall disease progression, often overemphasizing certain diagnostic results while overlooking other crucial clinical or auxiliary examination results. Effective development of clinical reasoning involves a continuous and reflective learning process in which trainees identify problems, formulate relevant questions, and implement solutions through repeated practice. Debriefing has shown great potential as an effective strategy to address these challenges and enhance clinical reasoning development.

The vast scientific and technological changes have dramatically impacted medical education. One such example is medical simulation, which has attracted significant attention across institutions. Medical simulation education utilizes advanced medical techniques and methodologies, emphasizing experiential learning in a clinical teaching paradigm. The immersion in a virtual clinical environment enhances learners’ cognitive and clinical skills, allowing repetitive practicing without causing patients safety issues, and effectively fostering and evaluating the competences of resident trainees (3). Simulations are aligned with the experiential learning theory proposed by Townsend Rob, etc., offering hands-on opportunities to learn through situational simulations. Subsequently, students learn by reflecting upon these practical experiences to enhance their clinical thinking. As delineated in the “Medical Simulation Dictionary” 2020 edition by the Society for Simulation in healthcare, structured debriefing is characterized as a formal reflective discussion following a simulation activity, and essential to accomplish the educational goals of the simulation.

The concept of debriefing has its origins in psychology, serving as a therapeutic intervention (4). It later gained prominence in military operations and aviation simulation training before being incorporated into medical simulation education. Fanning and colleagues from Stanford University, along with Cantrell from Villanova University, have effectively integrated simulation-based education into anesthetic crisis resource management courses and clinical nursing simulation training (5). The results of these medical simulation studies have been profoundly impactful, advocating for the routine inclusion of crisis event simulations followed by debriefing sessions. This has solidified the pivotal role of debriefing in medical simulation education, rendering it an indispensable component. It’s often regarded as the “heart and soul” of simulation instruction (6). Debriefing focused on specific thought processes and actions helps trainees to identify knowledge and communication gaps and analyze the outcome of those thoughts and actions. The process is overseen by an instructor, adhering to a student-centered, teacher-supported philosophy (7). This encourages deeper student contemplation and cultivates their clinical thought process. The general structure of most debriefing sessions focuses on participant reactions, followed by analysis, and ending with a summary of lessons learned. The two leading debriefing models include the Structured and Supported Debriefing Model and the Debriefing with Good Judgment Model (DwGJ) (8, 9). The DwGJ Model advocates the instructor fostering a relaxing ambiance, approaching learners with true curiosity and feedback, using the advocacy and inquiry technique, while avoiding the urge to “fix” learners, which is better suited the innate nature of learners. This debriefing session is usually organized into 3 phases: reactions, analysis, and summary (7).

In China, residency training program has experienced changes during the past several decades to cope with the dramatic increase in people’s needs of high-quality medical services, as well as the rapid development of medical science and technology. In December 2013, seven Chinese government ministries jointly launched the Guidelines for Standardized Resident Training (SRT). The year 2014 marks the beginning of SRT in China (10). The guidelines become compulsory nationally by 2020. The aim of resident training is to equip medical graduates with practical clinical skills and enable them to become application-oriented, multi-skilled professionals serving in the national health system. Different from previous guidelines, SRT is focused mainly on cultivating qualified practitioners rather than academics. Therefore, developing effective teaching approaches and leveraging the educational environment to cultivate residents with professional competencies has become a key responsibility of medical educators.

Previous research has sought to determine the optimal approach to integrating simulation-based education for maximizing learner benefits. Chen et al. conducted a randomized study of 42 dental residents and found that combining micro-lectures with debriefing significantly improved theoretical knowledge, practical performance, clinical reasoning, problem-solving, and learning satisfaction compared with traditional training, suggesting its potential for enhancing residency education quality (11). Similarly, another study comparing debriefing-based teaching with traditional methods among clinical medicine students reported higher clinical skill scores and improvements in independent learning and learning efficiency in the debriefing group (12). Kang et al. evaluated the impact of integrating TeamSTEPPS (team strategies and tools to enhance performance and patient safety) with debriefing in anesthesia crisis simulation teaching among 40 residents, and they found that this combined approach significantly improved simulation performance, teamwork perception, teamwork attitudes, and learning satisfaction compared with regular simulation teaching (13). These findings suggest that expanding research on debriefing methods and strategies with larger sample sizes could extend their application across diverse aspects of clinical education, including scenario-based training in diagnostics and surgery, as well as clinical activities such as history-taking, clinical reasoning training, bedside teaching, and difficult case discussions, thereby enhancing skill acquisition and advancing clinical medical education reform. Pertinent studies indicate that combining simulation teaching with Case-Based Learning (CBL) yields substantial educational benefits (14). However, the effectiveness of this combined approach for Chinese medical students remain unclear. This study aims to investigate the effects of employing the “debriefing with good judgment” technique combined with the CBL in training the clinical reasoning of trainee physicians in China’s standardized residency program.

## Methods

### Course introduction and participants

This study was designed and conducted as a randomized controlled educational trial. The sample size in the current study was determined by G*Power with referring to effect size reported in previous simulation-based education research (15). *A priori* sample size estimation was conducted using G*Power V3.1, and a medium-to-large effect size (Cohen’s *d* = 0.70) was assumed. With a two-tailed *α* = 0.05 and power (1 − *β*) = 0.80, the calculation indicated a minimum of 34 participants per group. From October 2023 to January 2024, a total of 70 resident physicians undergoing standardized training at the Clinical Skills Training Center of the First Affiliated Hospital of Jinan University, Guangzhou, China were recruited for this study. The participants were first-year trainees from multiple departments, including Internal Medicine, Surgery, Obstetrics and Gynecology, Pediatrics, Emergency Medicine, and Intensive Care Medicine. The participants from each department were randomly assigned to either a control group (*n* = 35) or a DwGJ group (*n* = 35). All participants were informed of the study’s objectives and provided with written informed consent. Both groups received the same theoretical instruction, delivered by experienced physicians in a shared classroom. The sessions covered essential clinical skills, including definitions, indications, contraindications, relevant anatomical knowledge, preoperative preparations, procedural steps, and precautions. The skill-based courses totaled 42 academic hours and spanned multiple disciplines. The content was aligned with the Accreditation Council for Graduate Medical Education (ACGME) advanced requirements for resident training (16). Each condition was framed through three case studies of varying complexity: elementary, intermediate, and advanced. The case assignment was based on the proficiency level of the resident trainees.

### Study design for the clinical skill practice

**Figure 1:**
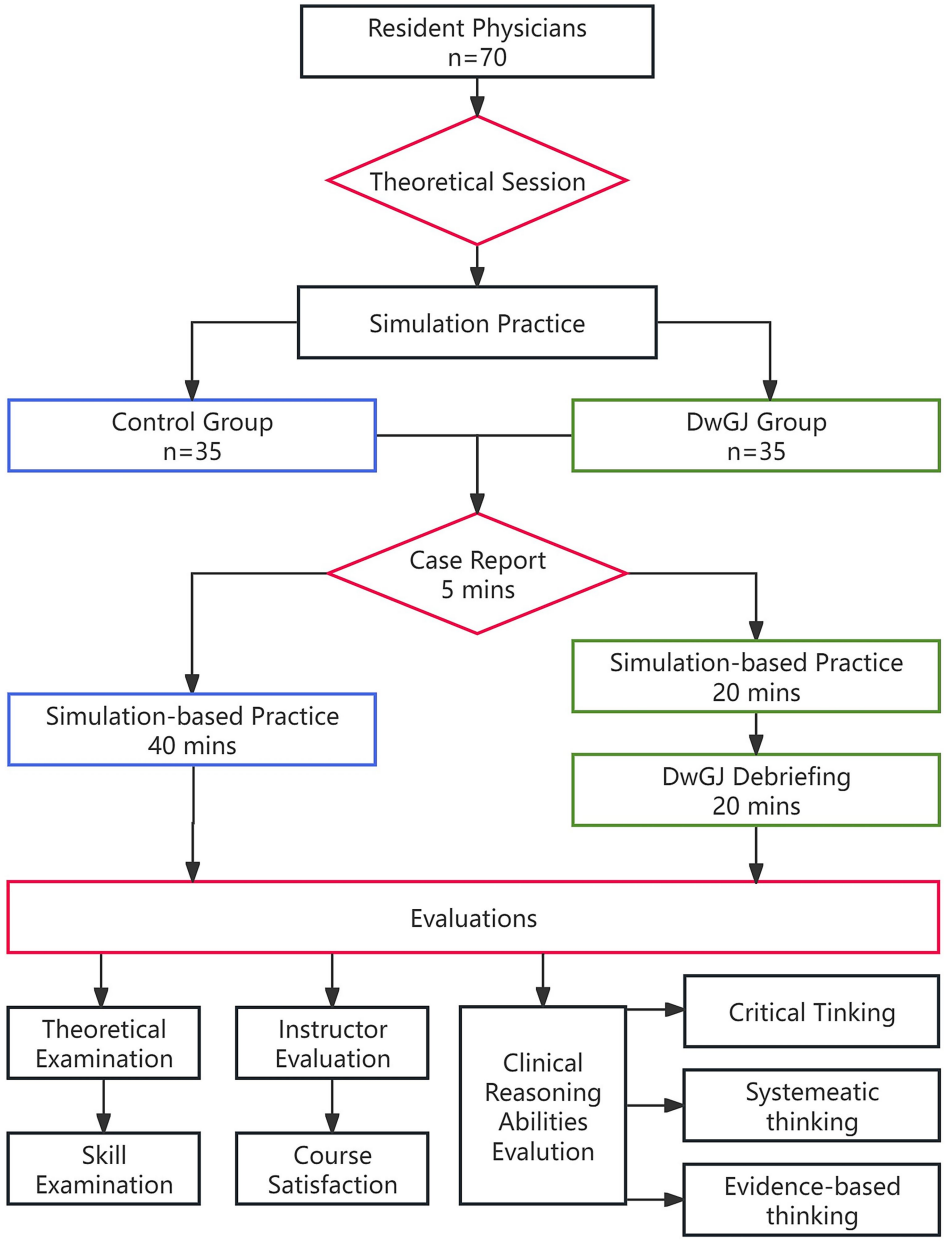
The flow chart illustrating the main steps of the study.

**Figure 2:**
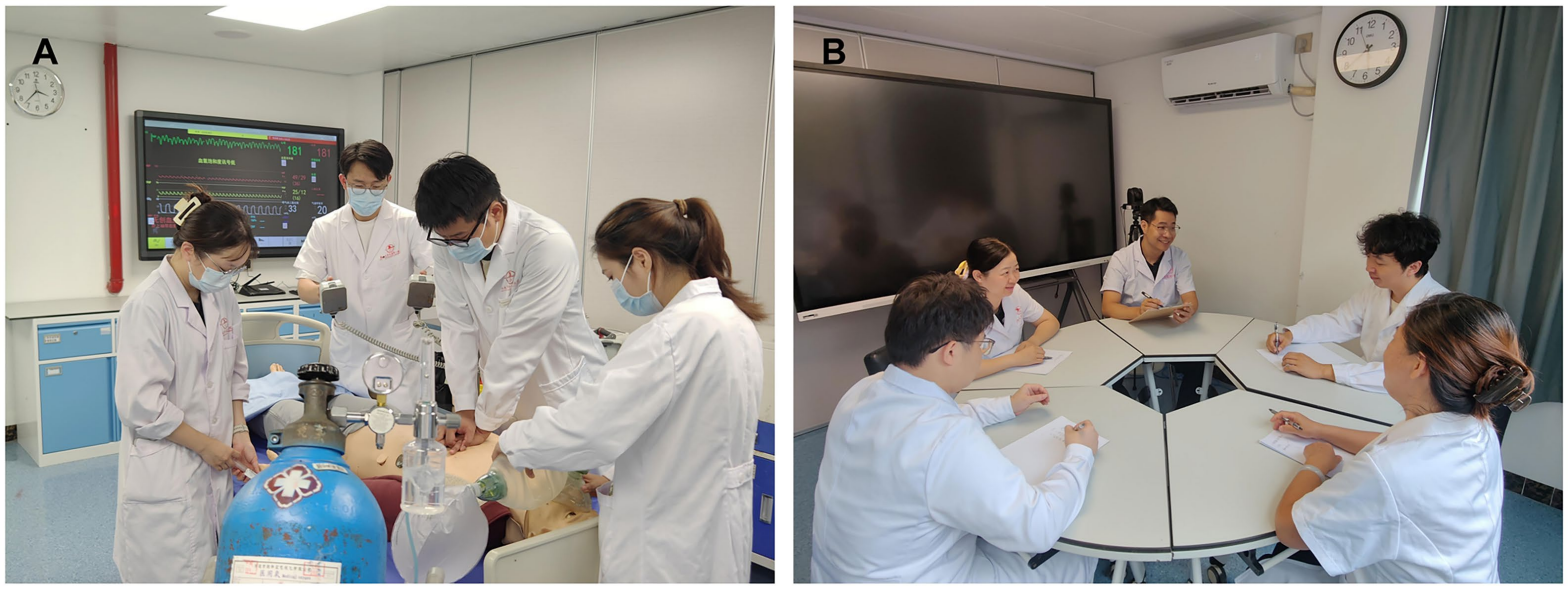
The simulation-based practice session. **(A)** The participants of the control group performed the clinical skills with instructor’s supervision for 40 min based on a simulated case scenario; **(B)** the participants of the DwGJ group performed clinical skills practice for 20 min based on a simulated case scenario, followed by a debriefing session focused on the issues identified during the clinical skills practice.

Following the completion of theoretical learning, the trainees from both group were divided into subgroups of four for simulation-based clinical procedures. Figure 1 is the diagram flow chart of the study. The control group followed the conventional CBL approach. Briefly, appropriate case studies were selected for classroom integration, a simulated on-site environment was created using elements such as the SimMan 3G simulator, ambient music, and video footage to replicate the real scenario (Figure 2A). As the case unfolded, residents were required to perform clinical procedures tailored to the case requirements, with learning in groups and rotating roles. Instructors provided prompt feedback, identifying areas for improvement, and providing corrective demonstrations as needed.

The DwGJ group received debriefing-based simulation integrated with CBL. The same clinical cases used in the control group were applied. Within each group, two members assumed hands-on roles, while the other two acted as observers, then rotated roles. Each 20-min hands-on session was followed by a 20-min DwGJ roundtable debriefing. In the initial phase, a member from the hands-on group recounted the case evolution, allowing peers to evaluate and share authentic feelings. The “Plus/Delta” strategy was employed. The subsequent phase involved analysis. The residents interspersed their contributions with “reflective” clinical thinking. They were encouraged to recognize both their shortcomings and strengths, and to delve into discussions surrounding the challenges they faced. The “Advocacy and Inquiry” were employed to ensure candid conversations. The instructor served as a facilitator, promptly identifying variations in residents’ procedures to guide deeper analysis. In the concluding phase, the instructor offered solutions to the identified challenges or shared experiential insights (Figure 2B). The total teaching hours were the same as those of the control group.

### Evaluation strategies

#### Theoretical examination and skills assessments

The assessment comprised a theoretical test and an Objective Structured Clinical Examination (OSCE) based skill evaluation. A total of 50 multiple choice questions were randomly selected from the question bank and distributed identically to both groups for the theoretical examination. The established question bank contained nearly one thousand questions contributed by faculty members from relevant departments. All questions in the bank were categorized by levels of difficult, moderate, and easy. The total score for the test was 100 points. The OSCE consisted of five stations designed to evaluate different dimensions of clinical competence: history taking, physical examination, clinical skills operations, clinical reasoning and case analysis, and medical documentation. The performance was evaluated by two examiners independently. For the clinical skills operation station, each participant was randomly assigned one of four established tasks of comparable difficulty: cardiopulmonary resuscitation, diagnostic curettage, thoracentesis, and on-site external fixation for fractures. The standard three phases of skills assessments: pre-procedure, intra-procedure, and post-procedure, were evaluated. Each station was scored out of 20, with a total score of 100 points. The final score was the average of the two examiners’ evaluations at each station, ensuring consistent and reliable assessment across participants.

#### Survey questionnaire

A questionnaire consisting of 47 items was distributed to the participants via Sojump (Ranxing, Changsha, China) after the course assessments (Supplementary Data 1). The questionnaire was adapted from the “Chinese Clinical Doctor Competency Model Construction and Application,” the “Chinese Clinical Doctor Competency Survey,” and previous publications (17, 18). It comprised two parts: (1) Instructor evaluations and course satisfaction (19 items), and (2) Clinical reasoning abilities, which included three dimensions: critical thinking skills (7 items), systematic thinking (11 items), and evidence-based thinking skills (7 items). Each item was rated on a five-Likert scale: excellent, good, average, poor very poor, which corresponds to 5, 4, 3, 2, and 1 score, respectively.

### Data analysis

All data were analyzed using SPSS 27.0. Categorical data was presented by frequency and percentage, with inter-group comparisons conducted using the Chi-squared test. Quantitative data were expressed as mean ± standard deviation, with the t-test employed for inter-group comparisons. The significant level was set at *α* = 0.05. Cronbach’s alpha coefficient was used to assess the reliability of the questionnaire.

## Results

### Demographic characteristics of the participants and study implementation

A total of 15 males and 20 females were included in the control group, with an average age of 26.09 ± 1.38 years. The experimental group comprised 14 males and 21 females, with an average age of 26.20 ± 1.53 years (Table 1). There was no significant difference in age distribution between the control and DwGJ group (*t* = −0.60, *p* = 0.55). The number of residents with prior clinical experience was 6 in the control group and 5 in the DwGJ group. The theoretical and skill-based admission examination scores were compared between the two groups, and no significant differences were observed (Theory: Control 360.29 ± 13.13 *vs* DwGJ 364.66 ± 8.67; *t* = −1.64, *p* = 0.11; Skill: Control 80.11 ± 4.66 *vs* DwGJ: 78.89 ± 4.55; *t* = 1.12, *p* = 0.27). Meanwhile, the distribution of the participants between the control and DwGJ groups did not differ significantly, with a Likelihood ratio of 0.84 (Monte Carlo Significance).

**Table 1:** Demographic characteristics of residents in the control and DwGJ groups.

Characteristic	Control group *n* = 35	DwGJ group *n* = 35	*p*
Age (*®X* ± SD)	26.09 ± 1.38	26.20 ± 1.53	0.55
Gender			
Male, *n* (%)	15 (42.86%)	20 (57.14%)	
Female, *n* (%)	14 (40.00%)	21 (60.00%)	
Prior clinical experience, *n* (%)	6 (17.14%)	5 (14.29%)	
Theoretical examination scores (*®X* ± SD)	360.29 ± 13.13	364.66 ± 8.67	0.11^1^
Skill examination scores (*®X* ± SD)	80.11 ± 4.66	78.89 ± 4.55	0.27^1^
Specialty, *n* (%)			0.84^2^
Internal medicine	11 (31.43%)	12 (34.29%)	
Surgery	10 (28.57%)	8 (22.86%)	
Obstetrics and gynecology	3 (8.57%)	6 (17.14%)	
Pediatrics	3 (8.57%)	4 (11.43%)	
Emergency medicine	4 (11.43%)	2 (5.71%)	
Intensive care medicine	4 (11.43%)	3 (8.57%)	

^1^Independent-samples *t*-test; ^2^Likelihood ratio (Monte Carlo Significance).

### Participants in the DwGJ group reported higher levels of course satisfaction

The post-course assessments comprised two components: evaluation of the instructors and evaluation of the course contents. No significant difference was observed between the DwGJ and control groups regarding satisfaction with the instructors’ teaching attitudes, with both groups expressing positive feedback. However, students in the DwGJ group demonstrated generally higher satisfaction with their learning outcomes. Additionally, their evaluations of the instructors’ effectiveness in promoting critical thinking were significantly higher than those of the control group (Table 2).

**Table 2:** Comparison of perceived learning outcomes between residents in the control and DwGJ groups (*®X* ± SD).

Items	Control group *n* = 35	DwGJ group *n* = 35	*t*	*p*
1. Understand and master theoretical knowledge.	3.34 ± 1.24	3.54 ± 0.92	−0.77	0.45
2. Master methods such as self-study and literature consultation to acquire new knowledge.	3.57 ± 0.74	4.37 ± 0.73	−4.55	<0.01
3. Master the knowledge content of relevant multiple disciplines.	4.09 ± 0.82	4.46 ± 0.74	−1.99	0.51
4. Categorize and integrate information and materials.	3.65 ± 0.64	4.54 ± 0.78	−5.19	<0.01
5. Improve clinical response capabilities.	3.71 ± 0.62	4.65 ± 0.63	−6.25	<0.01
6. Improve the ability to assess and operate clinical conditions.	3.69 ± 0.63	4.68 ± 0.63	−6.62	<0.01
7. Improve clinical thinking and judgment abilities.	3.74 ± 0.61	4.68 ± 0.58	−6.60	<0.01
8. Enhance humanistic concepts.	4.20 ± 0.72	4.40 ± 0.85	−1.06	0.29
9. Enhance emergency response capabilities and rescue capabilities.	3.77 ± 0.60	4.11 ± 0.93	−1.83	0.07
10. Improve teamwork ability.	3.74 ± 0.70	4.71 ± 0.58	−6.12	<0.01
11. Improve communication skills	3.71 ± 0.82	4.65 ± 0.63	−5.34	<0.01
12. Enhance learning interest.	3.74 ± 0.70	4.74 ± 0.56	−6.59	<0.01
13. Ability of creative thinking	3.66 ± 0.76	4.09 ± 0.89	−2.17	0.03
Total satisfaction	3.74 ± 0.46	4.43 ± 0.48	−6.15	<0.01

### DwGJ improved the participants’ academic performance

Following the completion of the course, both theoretical knowledge and practical skills were assessed. Theoretical test scores were significantly higher in the DwGJ group (64.40 ± 13.22) compared to the control group (52.34 ± 18.42; *p* < 0.01). Similarly, the DwGJ group outperformed the control group in practical skill assessments, with mean scores of 80.54 ± 7.40 and 72.32 ± 7.6, respectively (*p* < 0.01).

### DwGJ significantly enhanced the participants’ clinical reasoning abilities

The questionnaire used to assess clinical reasoning abilities included items evaluating critical thinking skills, systematic reasoning, and evidence-based thinking. The Cronbach’s alpha coefficient for these dimensions were 0,983, 0.984 and 0.965, respectively, all exceeding 0.9, which indicated excellent internal consistency and reliability. The Kaiser-Meyer-Olkin (KMO) values for each dimension were 0.925, 0.936, and 0.915, respectively. Bartlett’s Test of Sphericity was notably significant (*p* < 0.01) at the 0.01 level, which supported the construct validity of the questionnaire and suggested the three identified factors represent the underlying structure of clinical reasoning abilities. Following the DwGJ simulation-based feedback training, participants in DwGJ group consistently outperformed those in the control group across multiple dimensions of clinical reasoning, including self-reported critical thinking skills (Table 3), systematic thinking capabilities (Table 4), and evidence-based thinking (Table 5). Most of the differences were statistically significant (*p* < 0.05).

**Table 3:** Comparison of self-reported critical thinking skills between residents in the control and DwGJ groups(*®X* ± SD).

Items	Control group *n* = 35	DwGJ group *n* = 35	*t*	*p*
1. Seeking for truth.	4.34 ± 0.68	4.49 ± 0.74	−0.84	0.41
2. Open-mindedness.	3.82 ± 0.66	4.51 ± 0.70	−4.20	<0.01
3. Analytical skills.	4.40 ± 0.55	4.57 ± 0.70	−1.14	0.26
4. Systematic ability to work on problems in an organized, purposeful manner.	3.86 ± 0.65	4.51 ± 0.70	−4.07	<0.01
5. Self-confidence in critical thinking.	3.80 ± 0.63	4.48 ± 0.74	−4.16	<0.01
6. Intellectual curiosity.	4.37 ± 0.65	4.49 ± 0.74	−0.69	0.49
7. Cognitive maturity.	4.37 ± 0.60	4.43 ± 0.73	−0.36	0.72

**Table 4:** Comparison of self-reported systematic thinking skills between residents in the control and DwGJ groups (*®X* ± SD).

Items	Control group *n* = 35	DwGJ group *n* = 35	*t*	*p*
1. The degree of basic and clinical knowledge and the ability to recognize clinical signs and symptoms.	4.26 ± 0.70	4.51 ± 0.74	−1.49	0.14
2. Ability to obtain and recognize a variety of physical signs using proper examination techniques and the ability of the patient to cooperate.	3.68 ± 0.47	4.45 ± 0.78	−5.01	<0.01
3. Availability of auxiliary inspection: The ability to rationally utilize auxiliary inspection.	3.97 ± 0.75	4.46 ± 0.82	−2.60	0.012
4. History Taking: Detailed questioning and recording of the patient’s medical history.	4.06 ± 0.73	4.43 ± 0.70	−2.18	0.03
5. Disease observation: Accurate and timely observation of the patient’s disease changes.	3.77 ± 0.54	4.45 ± 0.78	−4.26	<0.01
6. When collecting patient information, different information can be automatically categorized inside your head.	3.54 ± 0.56	3.66 ± 0.84	−0.67	0.51
7. The ability of gather information and consider different perspectives when confused about a patient’s health issue.	4.11 ± 0.68	4.34 ± 0.84	−1.26	0.24
8. With the continuous presentation or introduction of the disease, I can regularly sort out the collected information and confirm my own ideas.	3.71 ± 0.51	4.40 ± 0.77	−4.35	<0.01
9. When a piece of information comes up and makes me aware of a possible health problem, it can often cause me to review the information I have previously gathered to see if it fits that health problem.	3.71 ± 0.51	4.40 ± 0.77	−4.35	<0.01
10. Ability to fully gain the patient’s trust and communicate with the patient with reasonable skill in obtaining the required information.	3.63 ± 0.55	3.69 ± 1.13	−0.27	0.79
11. The ability to summarize the medical history in a clear, concise, and organized manner, with accurate descriptions of symptoms and signs and appropriate use of language that can be accurately understood by the patient.	3.71 ± 0.57	4.34 ± 0.76	−3.89	<0.01

**Table 5:** Comparison of self-reported evidence-based thinking skills between residents in the control and DwGJ groups (*®X* ± SD).

Items	Control group *n* = 35	DwGJ group *n* = 35	*t*	*p*
1. Research ability.	3.94 ± 0.91	4.34 ± 0.80	−1.96	0.06
2. Knowledge of evidence-based medicine.	3.54 ± 0.61	4.28 ± 0.78	−4.41	<0.01
3. The ability to translate medical problems and disagreements encountered in clinical practice into clear, specific, and answerable questions prior to implementation.	4.00 ± 0.69	4.37 ± 0.73	−2.19	0.03
4. Ability to use databases of evidence-based medicine and the Internet for retrieval of information and evidence.	4.00 ± 0.73	4.34 ± 0.76	−1.92	0.06
5. Ability to critically evaluate the quality of the literature and evidence reviewed against existing evidence-based standards.	3.31 ± 0.71	4.22 ± 0.80	−5.00	<0.01
6. Ability to determine the validity of evidence.	3.48 ± 0.56	4.40 ± 0.77	−5.65	<0.01
7. The ability to effectively integrate the best available research evidence with your clinical experience to make it suitable for clinical use.	3.48 ± 0.61	4.40 ± 0.81	−5.32	<0.01

## Discussion

Simulation-based education is a clinical teaching approach grounded in experiential learning principles, as exemplified by Kolb’s experiential learning theory (19), and it utilizes medical simulation technologies to facilitate this process. Due to its effectiveness in cultivating and assessing healthcare professionals’ competencies in specific clinical scenarios, simulation education has attracted increasing attention at medical schools and hospitals worldwide. Scenario-based simulation teaching is a structured instructional method typically composed of three phases: briefing (pre-scenario orientation), facilitation (guided scenario progression), and debriefing (post-scenario reflection). Among these, debriefing is widely regarded as the most critical yet challenging component to master in simulation education.

A variety of debriefing models are employed in simulation-based teaching, including the Structured and Supported model, the 3D model, and the “Masonry” model (20, 21). To date, there is no conclusive evidence in the literature indicating which model is superior. Simulation instructors typically implement one of three primary debriefing approaches: judgmental debriefing, characterized by direct critique; non-judgmental debriefing, which avoids explicit evaluation; and DwGJ, a reflective and constructive strategy that combines honest feedback with psychological safety (22, 23). Each approach offers distinct benefits when applied appropriately. In this study, we adopted the DwGJ model, following its three established phase: reactions, analysis, and summary. Our results showed that when integrated with the CBL method, DwGJ led to significantly higher levels of learner perceived learning achievement, improved clinical reasoning, and better assessment scores compared to the conventional CBL approach, thereby offering greater educational benefits. Possible reasons include: the trainee creates concrete experiences through simulation and completes critical reflective observation by debriefing, thereby constructing a complete learning cycle (19); or the debriefing session promotes critical reflection on learners’ implicit cognitive frameworks, achieving a shift in perspective and thereby enhancing deeper clinical reasoning skills (24). Integrating both approaches represents not merely a superimposition of teaching activities, but rather a systematic optimization of the learning process and cognitive mechanisms guided by theory. In this trial, both the DwGJ and control groups obtained relatively low theoretical examination scores. This outcome was likely attributable to the higher difficulty level of the selected test items. Although the primary intervention of this study focused on the skills-practice component, relevant theoretical foundations, diagnostic reasoning, and differential diagnosis were frequently reinforced during the debriefing sessions. Therefore, the intervention may have contributed to improvements in both theoretical knowledge and practical performance.

The simulation-based CBL teaching model adopts a learner-centered approach, positioning instructors primarily as facilitators who guide students through realistic clinical scenarios (25). This approach effectively fosters active learning among residents and enhanced their intrinsic motivation to engage with the material. The debriefing phase is pivotal, as it largely determines the overall effectiveness of simulation-based instruction. Conducting debriefing sessions immediately after skill execution enables learners to promptly reflect on their performance, identify both strengths and areas for improvement, and engage in targeted self-assessment. Through collaboratively identifying issues and developing corrective strategies, learners deepen their self-awareness and enhance clinical reasoning abilities. This process not only improves learning outcomes but also fosters greater engagement and promotes clinical cognition.

Previous studies have reported that the DwGJ model is more effective in enhancing students’ clinical reasoning compared to both judgmental and non-judgmental debriefings approaches (22, 26, 27). In this study, we similarly found that the students in the DwGJ group reported significantly greater improvement in critical thinking and clinical judgment development than those in the control group, aligning with the findings of Vermeulen and others (17). We propose that the observed benefits in critical thinking are closely linked to the structure of simulation-based teaching. Instructors create a psychologically safe environment in which students are encouraged to make mistakes without fear of judgment (28, 29). This safe space allows students to make mistakes in the safe environment, internalize lessons, and accumulate experience, thereby better preparing them for similar scenarios in real clinical practice. Additionally, this process helps build students’ self-confidence, clinical thinking, decision-making, disease assessment capabilities, and technical proficiency (30).

This study found that evaluation of both systematic thinking and evidence-based thinking were significantly higher in the DwGJ group compared to the control group. Systematic thinking requires a solid foundation of clinical knowledge and thorough mental preparation (31). In this study, instructors intentionally guided learners to develop systematic thinking, especially by addressing the question: “Why is this procedure being performed, and what is its clinical purpose?” For example, residents who participated in the operation were asked to describe the patient’s clinical course, including personal information, chief complaint, current history, past medical history, personal and family history, birth history, physical examination results, and auxiliary test results. They were required to summarize, analyze, reason through, and articulate the rationale for the intervention. Through this complete feedback-driven practice loop, learners in the DwGJ group demonstrated marked improvement in systematic thinking and consolidation of clinical knowledge, resulting in enhanced diagnostic and treatment competency.

Furthermore, the combination of DwGJ-based debriefing and the CBL approach effectively stimulated learners to think through complex clinical problems. When faced with uncertainties in clinical scenarios, learners were able to consult the literature, reflect on current evidence, and improve their self-learning ability. Most participants demonstrated the ability to define clinical problems, retrieve and appraise evidence, apply it appropriately, and assess outcomes.

### Limitations

This study was conducted at a single-center, and the sample size was relatively small. Clinical reasoning is a complex skill to assess, and most evaluations in this study were self-reported and performed within a simulated instructional context, which limited the reproducibility and generalizability of the findings. Future studies should consider expanding the sample size, applying the DwGJ debriefing-based simulation teaching combined with the CBL approach across broader training populations and incorporation of more objective assessment tools. Additionally, the duration of some debriefing sessions was not strictly controlled, which occasionally limited the time available for trainees to practice specific technical skills. Therefore, when implementing this instructional approach in the future, it is important to ensure a well-structured allocation of teaching hours and optimize the design of review sessions. A further limitation was the shortage of qualified instructors, which posed a significant challenge during implementation. This issue could be addressed in future studies by enhancing faculty development programs and assigning multiple trained instructors to facilitate simultaneous sessions, thereby ensuring that all trainees have sufficient opportunities for hands-on practice.

## Conclusion

This study demonstrated that the DwGJ debriefing-based teaching method, when integrated with the CBL approach, provides benefits for resident trainees compared to the CBL method alone. This combined approach significantly enhances trainees’ satisfaction with the teaching process, as well as their perceived enhancements of clinical reasoning, systematic thinking, and evidence-based thinking abilities. These findings collectively suggest potential advantages of this combined teaching strategy in supporting resident development, which could be applied in other educational settings, such as bedside clinical instruction and the discussion of complex or challenging cases, to further explore its educational impact.

## Data Availability

The raw data supporting the conclusions of this article will be made available by the authors, without undue reservation.

## References

[1] Epstein RM Hundert EM. Defining and assessing professional competence. JAMA. (2002) 287:226–35. doi: 10.1001/jama.287.2.226, 11779266

[2] Ten Cate O. Competency-based postgraduate medical education: past, present and future. GMS J Med Educ. (2017) 34:Doc69. doi: 10.3205/zma001146, 29226237 PMC5704607

[3] Cheng A Eppich W Grant V Sherbino J Zendejas B Cook DA. Debriefing for technology-enhanced simulation: a systematic review and meta-analysis. Med Educ. (2014) 48:657–66. doi: 10.1111/medu.12432, 24909527

[4] Everly GS Boyle SH. Critical incident stress debriefing (CISD): a meta-analysis. Int J Emerg Ment Health. (1999) 1:165–8. 11232385

[5] Fanning RM Gaba DM. The role of debriefing in simulation-based learning. Simul Healthc. (2007) 2:115–25. doi: 10.1097/SIH.0b013e3180315539, 19088616

[6] Gardner R. Introduction to debriefing. Semin Perinatol. (2013) 37:166–74. doi: 10.1053/j.semperi.2013.02.008, 23721773

[7] Bowe SN Johnson K Puscas L. Facilitation and debriefing in simulation education. Otolaryngol Clin N Am. (2017) 50:989–1001. doi: 10.1016/j.otc.2017.05.009, 28822579

[8] Szyld D Rudolph JW. Debriefing with good judgment In: Levine AI DeMaria S Schwartz AD Sim AJ, editors. The comprehensive textbook of healthcare simulation. New York, NY: Springer (2013)

[9] Phrampus PE O’Donnell JM. Debriefing using a structured and supported approach In: Levine AI DeMaria S Schwartz AD Sim AJ, editors. The comprehensive textbook of healthcare simulation. New York, NY: Springer (2013)

[10] Liu Y Qiu L Xue Y Wu J Li N. China's standardized residency training in the context of public health: a controversial decade-signs of hope? Front Public Health. (2025) 13:1552075. doi: 10.3389/fpubh.2025.1552075, 40401061 PMC12092433

[11] Song J Zhang C Yan L Jiang C Zhou S Chen J. Applied research on micro-lecture combined with debriefing teaching method in standardizing resident training. Chin J Clin Lab Manag (Electron Ed). (2025) 13:122–8. doi: 10.3877/cma.j.issn.2095-5820.2025.02.010

[12] Yang W Li L Ji X Shu X Ruan M Gong J . The role of debriefing in clinical skill teaching and its application. Chin J Med Educ. (2020) 40:30–3. doi: 10.3760/cma.j.issn.1673-677X.2020.01.008

[13] Zhu B Han M Wang S Kang F. Application of TeamSTEPPS combined with debriefing in simulation teaching of anesthesia crisis management. Chin J Med Educ. (2024) 44:28–32. doi: 10.3760/cma.j.cn115259-20230210-00119

[14] Rudolph JW Simon R Raemer DB Eppich WJ. Debriefing as formative assessment: closing performance gaps in medical education. Acad Emerg Med. (2008) 15:1010–6. doi: 10.1111/j.1553-2712.2008.00248.x, 18945231

[15] Ha EH. Effects of peer-led debriefing using simulation with case-based learning: written vs. observed debriefing. Nurse Educ Today. (2020) 84:104249. doi: 10.1016/j.nedt.2019.104249, 31683133

[16] Swing SR Beeson MS Carraccio C Coburn M Iobst W Selden NR . Educational milestone development in the first 7 specialties to enter the next accreditation system. J Grad Med Educ. (2013) 5:98–106. doi: 10.4300/JGME-05-01-33, 24404235 PMC3613328

[17] Vermeulen J Buyl R D'Haenens F Swinnen E Stas L Gucciardo L . Midwifery students' satisfaction with perinatal simulation-based training. Women Birth. (2021) 34:554–62. doi: 10.1016/j.wombi.2020.12.006 33384256

[18] Zhang YP Liu WH Yan YT Zhang Y Wei HH Porr C. Developing student evidence-based practice questionnaire (S-EBPQ) for undergraduate nursing students: reliability and validity of a Chinese adaptation. J Eval Clin Pract. (2019) 25:536–42. doi: 10.1111/jep.12897, 29573062

[19] Kolb DA. Experiential learning: Experience as the source of learning and development. Englewood Cliffs, NJ: Prentice Hall (1984).

[20] Jaye P Thomas L Reedy G. 'The diamond': a structure for simulation debrief. Clin Teach. (2015) 12:171–5. doi: 10.1111/tct.12300, 26009951 PMC4497353

[21] Eppich W Cheng A. Promoting excellence and reflective learning in simulation (PEARLS): development and rationale for a blended approach to health care simulation debriefing. Simul Healthc. (2015) 10:106–15. doi: 10.1097/SIH.0000000000000072, 25710312

[22] Rudolph JW Simon R Rivard P Dufresne RL Raemer DB. Debriefing with good judgment: combining rigorous feedback with genuine inquiry. Anesthesiol Clin. (2007) 25:361–76. doi: 10.1016/j.anclin.2007.03.007, 17574196

[23] Rudolph JW Simon R Dufresne RL Raemer DB. There's no such thing as "nonjudgmental" debriefing: a theory and method for debriefing with good judgment. Simul Healthc. (2006) 1:49–55. doi: 10.1097/01266021-200600110-00006, 19088574

[24] Mezirow J. Transformative learning: Theory to practice. New directions for adult and continuing education, no 74 San Francisco: Jossey-Bass; (1997). p. 5–12

[25] Fey MK Roussin CJ Rudolph JW Morse KJ Palaganas JC Szyld D. Teaching, coaching, or debriefing with good judgment: a roadmap for implementing "with good judgment" across the SimZones. Adv Simul (Lond). (2022) 7:39. doi: 10.1186/s41077-022-00235-y, 36435851 PMC9701361

[26] Thompson R Sullivan S Campbell K Osman I Statz B Jung HS. Does a written tool to guide structured debriefing improve discourse? Implications for Interprofessional team simulation. J Surg Educ. (2018) 75:e240–5. doi: 10.1016/j.jsurg.2018.07.001, 30093336

[27] Sharara-Chami R Sabouneh R Zeineddine R Banat R Fayad J Lakissian Z. In situ simulation: an essential tool for safe preparedness for the COVID-19 pandemic. Simul Healthc. (2020) 15:303–9. doi: 10.1097/SIH.0000000000000504, 32910106

[28] Rudolph JW Raemer DB Simon R. Establishing a safe container for learning in simulation: the role of the presimulation briefing. Simul Healthc. (2014) 9:339–49. doi: 10.1097/SIH.0000000000000047, 25188485

[29] Kolbe M Eppich W Rudolph J Meguerdichian M Catena H Cripps A . Managing psychological safety in debriefings: a dynamic balancing act. BMJ Simul Technol Enhanc Learn. (2020) 6:164–71. doi: 10.1136/bmjstel-2019-000470, 35518370 PMC8936758

[30] Roh YS Jang KI Issenberg SB. Gender differences in psychological safety, academic safety, cognitive load, and debriefing satisfaction in simulation-based learning. Nurse Educ. (2022) 47:E109–13. doi: 10.1097/NNE.0000000000001179, 35324496

[31] Kolbe M Marty A Seelandt J Grande B. How to debrief teamwork interactions: using circular questions to explore and change team interaction patterns. Adv Simul (Lond). (2016) 1:29. doi: 10.1186/s41077-016-0029-7, 29449998 PMC5806384

