# Supplementary material for: A combined approach of simulation-based “debriefing with good judgment” and case-based learning to enhance clinical thinking in Chinese residents
Source: Front Public Health. 2026 Feb 4;13:1718961. doi: 10.3389/fpubh.2025.1718961 (PMC12913574; doi:10.3389/fpubh.2025.1718961)
Supplement: Supplementary file 1 [file Table_1.DOCX]

**JNU First Affiliated Hospital Scenario Simulation Course Feedback (Student) Survey**

Dear fellow students: Your responses will be kept strictly confidential and used only for the purposes of this research. All data will be anonymized so that individuals cannot be identified.

**Basic Information**

Graduate: Resident Physician

Gender: male /female

Age:

Prior Work Experience Before Residency: Yes /No

Please read the following questions carefully and select your most genuine feedback. Thank you!

**I. Evaluation of the Instructors (Recognition: Yes/No)**

1. Teaching Responsibility : The instructor cares about students, focuses on teaching tasks, and monitors learning progress. [Single Choice]

-----------------------------------------------------------------------

○ Yes

○ No

-----------------------------------------------------------------------

2. Teaching Awareness : Demonstrates enthusiasm, actively answers questions, and exhibits strong teaching commitment.

-----------------------------------------------------------------------

○ Yes

○ No

-----------------------------------------------------------------------

3. Teaching Expression : Well-organized language, appropriate emphasis, and clarity without ambiguity.

-----------------------------------------------------------------------

○ Yes

○ No

-----------------------------------------------------------------------

4. Knowledge Proficiency : Solid expertise, familiar with key and challenging topics.

-----------------------------------------------------------------------

○ Yes

○ No

-----------------------------------------------------------------------

5. Communication Skills : Actively listens to student feedback and maintains effective interaction.

-----------------------------------------------------------------------

○ Yes

○ No

-----------------------------------------------------------------------

6. Teaching Methods : Integrates theory with clinical cases, employs diverse teaching approaches, and encourages critical thinking.

-----------------------------------------------------------------------

○ Yes

○ No

-----------------------------------------------------------------------

**II. Course Satisfaction**

1. Understanding Theoretical Knowledge

-----------------------------------------------------------------------

○ Excellent

○ Good

○ Average

○ Poor

○ Very Poor

-----------------------------------------------------------------------

2. Mastery of Self-Learning and Literature Review Methods

-----------------------------------------------------------------------

○ Excellent

○ Good

○ Average

○ Poor

○ Very Poor

-----------------------------------------------------------------------

3. Integration of Multidisciplinary Knowledge

-----------------------------------------------------------------------

○ Excellent

○ Good

○ Average

○ Poor

○ Very Poor

-----------------------------------------------------------------------

4. Information Organization and Synthesis

-----------------------------------------------------------------------

○ Excellent

○ Good

○ Average

○ Poor

○ Very Poor

-----------------------------------------------------------------------

5. Improvement in Clinical Response Capability

-----------------------------------------------------------------------

○ Excellent

○ Good

○ Average

○ Poor

○ Very Poor

-----------------------------------------------------------------------

6. Enhancement in Clinical Assessment and Procedural Skills

-----------------------------------------------------------------------

○ Excellent

○ Good

○ Average

○ Poor

○ Very Poor

-----------------------------------------------------------------------

7. Development of Clinical Reasoning and Judgment

-----------------------------------------------------------------------

○ Excellent

○ Good

○ Average

○ Poor

○ Very Poor

-----------------------------------------------------------------------

8. Strengthening Humanistic Values

-----------------------------------------------------------------------

○ Excellent

○ Good

○ Average

○ Poor

○ Very Poor

-----------------------------------------------------------------------

9. Improvement in Emergency Response and Resuscitation Skills

-----------------------------------------------------------------------

○ Excellent

○ Good

○ Average

○ Poor

○ Very Poor

-----------------------------------------------------------------------

10. Enhancement of Team Collaboration

-----------------------------------------------------------------------

○ Excellent

○ Good

○ Average

○ Poor

○ Very Poor

-----------------------------------------------------------------------

11. Advancement in Communication Skills

-----------------------------------------------------------------------

○ Excellent

○ Good

○ Average

○ Poor

○ Very Poor

-----------------------------------------------------------------------

12. Increased Learning Interest

-----------------------------------------------------------------------

○ Excellent

○ Good

○ Average

○ Poor

○ Very Poor

-----------------------------------------------------------------------

13. Cultivation of Innovative Thinking

-----------------------------------------------------------------------

○ Excellent

○ Good

○ Average

○ Poor

○ Very Poor

--------------------------------------------------------------------------

**III. Self-Assessment of Clinical Reasoning Abilities**

**A. Critical Thinking Skills**

1. Quest for Truth: Maintains a sincere and objective attitude in seeking knowledge, even if findings contradict personal beliefs or interests.

-----------------------------------------------------------------------

○ Excellent

○ Good

○ Average

○ Poor

○ Very Poor

-----------------------------------------------------------------------

2. Open-Mindedness: Tolerates diverse opinions and guards against personal biases. [Single Choice]

-----------------------------------------------------------------------

○ Excellent

○ Good

○ Average

○ Poor

○ Very Poor

-----------------------------------------------------------------------

3. Analytical Ability: Identifies problems, interprets issues, and predicts outcomes using evidence and reasoning. [Single Choice]

-----------------------------------------------------------------------

○ Excellent

○ Good

○ Average

○ Poor

○ Very Poor

-----------------------------------------------------------------------

4. Systematic Approach: Addresses problems with organized and goal-oriented efforts. [Single Choice]

-----------------------------------------------------------------------

○ Excellent

○ Good

○ Average

○ Poor

○ Very Poor

-----------------------------------------------------------------------

5. Confidence in Critical Thinking: Trusts in personal rational analysis capabilities. [Single Choice]

-----------------------------------------------------------------------

○ Excellent

○ Good

○ Average

○ Poor

○ Very Poor

-----------------------------------------------------------------------

6. Intellectual Curiosity: Pursues knowledge passionately, even without immediate practical value. [Single Choice]

-----------------------------------------------------------------------

○ Excellent

○ Good

○ Average

○ Poor

○ Very Poor

-----------------------------------------------------------------------

7. Cognitive Maturity: Makes prudent judgments, revises decisions as needed, and acknowledges the necessity of interim solutions.

-----------------------------------------------------------------------

○ Excellent

○ Good

○ Average

○ Poor

○ Very Poor

-----------------------------------------------------------------------

1. **Systematic Thinking Skills**

1. Mastery of Foundational and Clinical Knowledge : Ability to recognize clinical symptoms and signs.

-----------------------------------------------------------------------

○ Excellent

○ Good

○ Average

○ Poor

○ Very Poor

-----------------------------------------------------------------------

2. Physical Examination Skills: Performs accurate examinations and ensures patient cooperation.

-----------------------------------------------------------------------

○ Excellent

○ Good

○ Average

○ Poor

○ Very Poor

-----------------------------------------------------------------------

3. Utilization of Diagnostic Tests: Appropriately employs auxiliary examinations.

-----------------------------------------------------------------------

○ Excellent

○ Good

○ Average

○ Poor

○ Very Poor

-----------------------------------------------------------------------

4. History Taking: Conducts thorough and detailed patient interviews.

-----------------------------------------------------------------------

○ Excellent

○ Good

○ Average

○ Poor

○ Very Poor

-----------------------------------------------------------------------

5. Patient Monitoring: Accurately and promptly observes changes in patient condition.

-----------------------------------------------------------------------

○ Excellent

○ Good

○ Average

○ Poor

○ Very Poor

-----------------------------------------------------------------------

6. Information Categorization: Automatically classifies data during patient assessment.

-----------------------------------------------------------------------

○ Excellent

○ Good

○ Average

○ Poor

○ Very Poor

-----------------------------------------------------------------------

7. Adaptive Data Collection: Persistently gathers and evaluates information from multiple perspectives.

-----------------------------------------------------------------------

○ Excellent

○ Good

○ Average

○ Poor

○ Very Poor

-----------------------------------------------------------------------

8. Dynamic Information Integration: Regularly synthesizes new data with existing hypotheses.

-----------------------------------------------------------------------

○ Excellent

○ Good

○ Average

○ Poor

○ Very Poor

-----------------------------------------------------------------------

9. Retrospective Analysis: Re-examines prior data when new insights emerge.

-----------------------------------------------------------------------

○ Excellent

○ Good

○ Average

○ Poor

○ Very Poor

-----------------------------------------------------------------------

10. Patient Trust and Communication: Establishes rapport and obtains essential information.

-----------------------------------------------------------------------

○ Excellent

○ Good

○ Average

○ Poor

○ Very Poor

-----------------------------------------------------------------------

11. Clear Case Summarization : Accurately documents history and communicates findings effectively.

-----------------------------------------------------------------------

○ Excellent

○ Good

○ Average

○ Poor

○ Very Poor

-----------------------------------------------------------------------

**C. Evidence-Based Thinking Skills**

1. Research Competency

-----------------------------------------------------------------------

○ Excellent

○ Good

○ Average

○ Poor

○ Very Poor

-----------------------------------------------------------------------

2. Awareness of Evidence-Based Medicine

-----------------------------------------------------------------------

○ Excellent

○ Good

○ Average

○ Poor

○ Very Poor

-----------------------------------------------------------------------

3. Formulating Answerable Clinical Questions

-----------------------------------------------------------------------

○ Excellent

○ Good

○ Average

○ Poor

○ Very Poor

-----------------------------------------------------------------------

4. Searching Evidence via Databases/Internet

-----------------------------------------------------------------------

○ Excellent

○ Good

○ Average

○ Poor

○ Very Poor

-----------------------------------------------------------------------

5. Critical Appraisal of Literature Quality

-----------------------------------------------------------------------

○ Excellent

○ Good

○ Average

○ Poor

○ Very Poor

-----------------------------------------------------------------------

6. Assessing Evidence Validity

-----------------------------------------------------------------------

○ Excellent

○ Good

○ Average

○ Poor

○ Very Poor

-----------------------------------------------------------------------

7. Integrating Evidence with Clinical Experience

-----------------------------------------------------------------------

○ Excellent

○ Good

○ Average

○ Poor

○ Very Poor

-----------------------------------------------------------------------
