# Supplementary material for: A combined approach of simulation-based “debriefing with good judgment” and case-based learning to enhance clinical thinking in Chinese residents
Source: Front Public Health. 2026 Feb 4;13:1718961. doi: 10.3389/fpubh.2025.1718961 (PMC12913574; doi:10.3389/fpubh.2025.1718961)
Supplement: Supplementary file 2 [file Table_2.DOCX]

| **Cardiopulmonary Resuscitation** | | | | | |
| --- | --- | --- | --- | --- | --- |
| **Candidate:** | | **Exam registration number:** | **Examiner:** | | |
| **Project** | **Content** | | | **Full marks**  **(point)** | **Score** |
| Preparation  (20 points) | Assess the safety of the environment, slap the patient's shoulder to assess consciousness, call for help and activate the emergency response system, palpate the carotid pulse, and observe the thorax for rise and fall to assess breathing. | | | 10.0 |  |
|  | Have the patient lie on their back on a flat surface, quickly unbutton their clothes, and loosen their belt. | | | 10.0 |  |
| Operation  (65 points) | The doctor kneels beside the patient, placing the heel of both palms on the junction of the middle and lower third of the sternum, with fingers raised to avoid touching the chest wall. | | | 10.0 |  |
|  | The doctor straightens the elbow and applies vertical downward pressure with body weight until the sternum sinks 5-6 cm. Immediately release the pressure without lifting the palm from the area, allowing full thoracic recoil. | | | 10.0 |  |
|  | Press frequency 100 ~ 120 times/min. | | | 5.0 |  |
|  | Chin Lift Technique: Lift the patient's jaw to open the airway by tilting the head backward. Use one hand to press the patient's forehead and maintain the head tilt, aligning the chin-earlobe line vertically with the ground. Simultaneously, lift the chin upward with the other hand. Clear secretions and foreign objects from the mouth and nose to ensure airway patency. | | | 5.0 |  |
|  | The doctor pinches the patient's nostrils with thumb and index finger, inhales, and then blows deeply and quickly into the patient's mouth, tightly pressing the lips together and completely covering them. | | | 5.0 |  |
|  | Sustain 1-second breaths (500-600ml) to elevate the patient's chest. | | | 5.0 |  |
|  | Keep the patient's mouth open and release the fingers pinning the nose, then assess chest recovery before performing the next breath. | | | 5.0 |  |
|  | One cycle consists of 30 chest compression followed by 2 artificial respiration. | | | 7.5 |  |
|  | Complete 5 cycles (2.5 points deducted for each cycle missed until 5.0 points are deducted). | | | 5.0 |  |
|  | Assessing recovery effectiveness (any two of the following five indicators are sufficient: observation of major artery pulsation, pupillary light reflex, consciousness, spontaneous breathing, and skin color). | | | 5.0 |  |
|  | After the operation, inform the patient's family of the first aid results and the next steps according to the condition. | | | 2.5 |  |
| Medical humanities literacy  (15 points) | Dignified demeanor, poised manner, and neat attire (work uniform). | | | 2.5 |  |
|  | Standardized and meticulous actions during operation (2.5 points), demonstrating care for the person being assisted (2.5 points), and attention to privacy protection (2.5 points). | | | 7.5 |  |
|  | Take the initiative to introduce yourself (2.5 points), with civilized language and amiable attitude (2.5 points). | | | 5.0 |  |
| Total | | | | 100 |  |

| **Examiner signature:** | **Signature of the person in charge:** | **Date:** |
| --- | --- | --- |

| **On-site external fixation for fractures** | | | | | |
| --- | --- | --- | --- | --- | --- |
|  | | | | | |
| **Candidate:** | | **Exam registration number:** | **Examiner:** | | |
| **Project** | **Content** | | | **Full marks**  **(point)** | **Score** |
| Preparation  (20 points) | Check the patient's vital signs (oral). | | | 5.0 |  |
|  | Examine the affected limb: expose the left upper arm to assess the wound and evaluate blood supply and function of the left hand. | | | 5.0 |  |
|  | Prepare sterile dressings, bandages and triangular towels. | | | 5.0 |  |
|  | Inform the patient of the purpose of bandaging and fixation and obtain the patient's cooperation to relieve anxiety and tension. | | | 5.0 |  |
| Operation  (65 points) | Expose the wound fully and remove dirt and foreign bodies around the wound. | | | 5.0 |  |
|  | Cover the wound with sterile gauze or cotton pad and apply moderate pressure dressing. | | | 10.0 |  |
|  | Fold the triangular scarf into a swallowtail style | | | 10.0 |  |
|  | Position the center of the triangular scarf at the middle-lower third of the left forearm. | | | 10.0 |  |
|  | Tie the ends of the triangular scarf behind the neck, suspend the forearms in front of the chest, and keep the elbows at a 90-degree angle. | | | 15.0 |  |
|  | Secure the left shoulder and elbow joints to the chest wall by wrapping a triangular scarf around the left upper arm and tying it under the right armpit. | | | 10.0 |  |
|  | Inform the patient of relevant precautions after the operation. | | | 5.0 |  |
| Medical humanities literacy  (15 points) | Dignified demeanor, poised manner, and neat attire (work uniform). | | | 2.5 |  |
|  | Standardized and meticulous actions during operation (2.5 points), demonstrating care for the person being assisted (2.5 points), and attention to privacy protection (2.5 points). | | | 7.5 |  |
|  | Take the initiative to introduce yourself (2.5 points), with civilized language and amiable attitude (2.5 points). | | | 5.0 |  |
| Total | | | | 100 |  |

| **Examiner signature:** | **Signature of the person in charge:** | **Date:** |
| --- | --- | --- |

| **Segmental curettage** | | | |
| --- | --- | --- | --- |
|  |  |  | |
| **Candidate: Exam registration number： Examiner:** | | | |
| **Project** | **Content** | **Full marks**  **(point)** | **Score** |
| Surgical procedure（70 points） | Disinfect the vulva and the vagina twice. | 5 |  |
|  | Open the curettage kit, lay out the sterile drape, and arrange the instruments. | 5 |  |
|  | Bimanual examination to check the position, size, tenderness, and adnexa of the uterus. | 8 |  |
|  | Change gloves. | 2 |  |
|  | Insert the speculum and disinfect the cervix twice. | 5 |  |
|  | The cervical forceps clamp the anterior lip of the cervix. | 5 |  |
|  | Use a curette to scrape the cervical canal, examine the shape of the tissue removed, and place it on gauze. | 5 |  |
|  | Probe technique (holding the probe, direction, reading the scale). | 5 |  |
|  | Curettage of the uterine cavity (technique, direction, depth, force when using the curette, and examination of tissue characteristics). | 20 |  |
|  | Check the characteristics of the scraped endometrium and place it on another piece of gauze. | 5 |  |
|  | Monitor bleeding and stop the bleeding. | 5 |  |
| Postoperative Instructions  (20 points) | Medical advice: Due to significant bleeding, follow-up is needed, rest, take anti-inflammatory treatment, abstain from sexual activity, and return for pathology review in one week. | 10 |  |
|  | Answer the purpose of the surgery (diagnosis, hemostasis). | 10 |  |
| Overall  (10 points) | Proficiency in operations, and humanistic care. | 10 |  |
| Total | | 100 |  |
| **Examiner signature:** | **Signature of the person in charge:** | **Date:** | |

| **Thoracentesis** |
| --- |
|  |

| **Candidate:** | | **Exam registration number:** | **Examiner:** | | |
| --- | --- | --- | --- | --- | --- |
| **Project** | **Content** | | | **Full Marks**  **(points)** | **Score** |
| Preparation  (20 points) | (1) Doctors wear hats and masks (hair and nose exposed), and wash hands (oral instruction). | | | 5.0 |  |
|  | (2) Inform the patient and family about the purpose and precautions of the procedure, obtain the patient's cooperation, and sign the informed consent form. | | | 5.0 |  |
|  | (3) Equipment required: thoracentesis kit, iodine tincture, cotton swabs, sterile gloves, adhesive tape, 2% lidocaine injection solution, 10ml and 50ml syringes, test tubes, etc. | | | 5.0 |  |
|  | (4) The patient sits facing the chair back, with both forearms resting on the back and the forehead resting on the forearms. | | | 5.0 |  |
| Operation  (65 points) | (1) Perform chest percussion by selecting one of the commonly used puncture sites in the sitting position and locating it on the body surface (7th-8th intercostal space along the left subscapular or posterior axillary line). | | | 5.0 |  |
|  | (2) Routine skin disinfection: Disinfect the puncture site three times from inside to outside, ensuring proper disinfection method and coverage. | | | 5.0 |  |
|  | (3) Wear sterile gloves (5.0 points); lay a sterile drape (5.0 points). | | | 10.0 |  |
|  | (4) Administer 2% lidocaine injection in a layered infiltration manner from the puncture site to the parietal pleura. | | | 5.0 |  |
|  | (5) Use a hemostat to clamp the rubber tube connected to the needle hub. | | | 5.0 |  |
|  | (6) With the left hand, position the skin at the puncture site using the index and middle fingers (5.0 points); with the right hand, insert the needle vertically from the lower edge of the next rib under local anesthesia. Once the needle feels the breakthrough, attach the syringe to the rubber tube's tail end. Then release the hemostat and hand it to the assistant (examiner), instructing them to use the hemostat to secure the needle (10.0 points). | | | 15.0 |  |
|  | (7) Slowly aspirate the accumulated fluid using a syringe (5.0 points); after collecting the specimen, instruct the assistant (examiner) to clamp the rubber tube with a hemostat (5.0 points). | | | 10.0 |  |
|  | (8) Withdraw the needle and apply pressure to the puncture site (2.5 points); disinfect the site, cover with sterile gauze, and secure with adhesive tape; send the specimen for testing (5.0 points). | | | 7.5 |  |
|  | (9) Organize the items and inform the patient and family members of the relevant precautions. | | | 2.5 |  |
| Medical humanities literacy  (15 points) | (1) Dignified demeanor, poised manner, and neat attire (work uniform). | | | 2.5 |  |
|  | (2) Standardized and meticulous actions during operation (2.5 points), demonstrating care for the person being assisted (2.5 points), and attention to privacy protection (2.5 points). | | | 7.5 |  |
|  | (3) Take the initiative to introduce yourself (2.5 points), with civilized language and amiable attitude (2.5 points). | | | 5.0 |  |
| Total | | | | 100 |  |

| **Examiner signature:** | | **Signature of the person in charge:** | | **Date:** | | |
| --- | --- | --- | --- | --- | --- | --- |
|  | |  | |  |  |  |
